# Is painful temporomandibular disorder a real headache for many patients?

**DOI:** 10.1038/s41415-024-7178-1

**Published:** 2024-03-22

**Authors:** Pankaew Yakkaphan, Leigh-Ann Elias, Priya Thimma Ravindranath, Tara Renton

**Affiliations:** 4141549005001 Faculty of Dentistry, Oral and Craniofacial Science, King´s College London, London, UK; Faculty of Dentistry, Prince of Songkla University, Songkhla, Thailand; 4141549005002 Orofacial Pain Service, Department of Oral Surgery, King´s College Hospital NHS Foundation Trust, London, UK; 4141549005003 Faculty of Dentistry, Oral and Craniofacial Science, King´s College London, London, UK; Orofacial Pain Service, Department of Oral Surgery, King´s College Hospital NHS Foundation Trust, London, UK

## Abstract

Temporomandibular disorders (TMDs) and primary headaches are common pain conditions and often co-exist. TMD classification includes the term ‘headache secondary to TMD' but this term does not acknowledge the likelihood that primary headache pathophysiology underpins headache causing painful TMD signs and symptoms in many patients. The two disorders have a complex link and we do not fully understand their interrelationship. However, growing evidence shows a significant association between the two disorders. This article reviews the possible connection between temporomandibular disorders and primary headaches, specifically migraine, both anatomically and pathogenetically.

## Key points

- The co-existence of temporomandibular disorders (TMDs) and primary headaches implies a potential connection in their underlying mechanisms.
- Diagnosis and treatment can be challenging due to the complex interrelationship between TMD and primary headaches, particularly migraines, underscoring the need for comprehensive pain history.
- Some individuals might experience painful TMD signs and symptoms as a result of an underlying headache disorder.

## Introduction

Temporomandibular disorders (TMDs) is an umbrella term for a heterogeneous condition of musculoskeletal disorders involving pain and/or functional limitations in the masticatory muscles, temporomandibular joints (TMJ) and associated structures in orofacial region.^1^ The condition is considered as the most common cause of non-dental orofacial pain and as the second most commonly occurring musculoskeletal condition.^2^ Approximately 40-70% of the general population experience some symptoms and signs of TMD.^3^^,^^4^^,^^5^ These conditions are found more often in women and commonly appears between the ages of 20-50 years.^6^^,^^7^A number of pain conditions has been associated with TMD, such as chronic fatigue syndrome, fibromyalgia, headaches, irritable bowel syndrome and chronic back pain.^8^^,^^9^ The diagnosis and classification of TMD have improved following the publication of the comprehensive and newly recommended Diagnostic Criteria for Temporomandibular Disorders (DC/TMD)^10^ and the International Classification of Orofacial Pain (ICOP).^11^

The two classification systems broadly classify TMD as muscle-related (or myogenous pain, including temporalis and masseter muscle pain) and joint-related problems that may cause pain (disc-displacement with and without reduction, subluxation and inflammatory change of the joint). TMDs, by definition, do not include other pathology, for example, neoplasia, infection or trauma. These classifications are outlined in Appendix 1.

Migraine is a subtype of primary headache condition manifested by a familial paroxysmal neurological disorder characterised by spontaneous or triggered headache attacks variably associated with autonomic disturbances, such as nausea, increased sensitivity to external stimuli (photophobia, phonophobia), and, less commonly, hemiparesis or aphasia.^12^ Migraine attacks usually last 4-72 hours.^12^ Migraine has a significant impact on the individual's quality of life and is ranked by the World Health Organisation as one of the 20 most debilitating diseases in the world.^13^ The best recognised diagnostic criteria for migraine is the third edition of International Classification of Headache Disorders (ICHD-3)^12^ (Appendix 2).

TMD and migraine are both characterised by pain in the head and/or face, and both conditions are more common in women, especially those of childbearing age.^14^^,^^15^^,^^16^ Although they are two completely different disorders, overlap is common, often leading to misdiagnosis or underdiagnosis of one or other condition. Several studies have explored prevalence of the comorbidity and relationship between migraine and TMD (Table 1).^17^^,^^18^^,^^19^^,^^20^^,^^21^^,^^22^^,^^23^^,^^24^^,^^25^^,^^26^^,^^27^^,^^28^^,^^29^^,^^30^^,^^31^^,^^32^^,^^33^ From these previous studies, it is sensible to suggest that TMD and migraine may have bidirectional association. TMD may be the original cause of headaches and may exacerbate and worsen existing headaches. Migraine headache may well trigger and aggravate TMD, or these two conditions may be comorbid. This review aims to explore the relationship between TMD and migraine, focusing on anatomical and pathophysiological aspects.

**Table 1:** Summary of studies on association and prevalence of TMD comorbid migraine

	Author, year, country	Study design	Association between TMD and migraine	Prevalence of TMD comorbid migraine
1	Ballegaard *et al*., 2008, Denmark^17^	Cross-sectional	-	Prevalence of TMD in migraine with and without aura was 53.3%
2	Gonçalves, 2009, Brazil^18^	Cross-sectional study	Prevalent ratio (PR) of TMD symptoms in migraine was 2.10	Prevalence of TMD in migraine was 58.2%
3	Barbosa *et al*., 2010, Brazil^19^	Case-control	Participants with episodic migraine (EM) are likely to have tenderness in the masticatory muscles with odd ratio (OR) = 3.0, chronic migraine (CM) had OR = 6.9. For tenderness in the TMJ, EM reported OR = 4.7), CM had OR = 4.8	83.87% of EM and 94.28% of CM presented at least one TMD sign
4	Franco *et al*., 2010, Brazil^20^	Case-control	TMD samples reported higher risk for any headache (OR = 7.05), and for migraine (OR = 2.76)	Prevalence of TMD in migraine was 55.26%
5	Gonçalves *et al*., 2010, Brazil^21^	Cross-sectional	TMD pain was increased in migraine patients (PR = 5.3)	Prevalence of TMD in migraine was 32.33%
6	Gonçalves *et al*., 2011, Brazil^22^	Cross-sectional	Individuals with myofascial TMD were significantly more likely to have migraine (risk ratio [RR] = 4.4)	Prevalence of TMD in migraine was 35.22%
7	Hoffmann *et al*., 2011, USA^23^	Case-control	Before TMD existing, 20% of TMD samples reported migraine. After TMD onset, the prevalence of migraine raised to 54%	Prevalence of migraine in TMD group was 50%
8	Gonçalves *et al*., 2013, Brazil^24^	Cross-sectional	Risk for TMD, EM had OR = 3.15 and CM showed OR = 3.97	Overall (EM + CM): TMD presented 88.52%. EM 86.8%, CM = 91.3%
9	Morais *et al*., 2015, Brazil^25^	Cross-sectional study	-	TMD prevalence in migraine was 71.34%
10	Dahan *et al*., 2016, Canada^26^	Cross-sectional	Self-reported migraine patients were likely to have myofascial TMD than non-myofascial TMD (OR = 3.00)	All TMD groups reported migraine: 46.11%. Myofascial TMD reported migraine: 54.55%. Non-myofascial TMD reported migraine: 28.81%
11	Florencio *et al*., 2017, Brazil^27^	Cross-sectional study	EM were more likely to have TMD with RR = 1.77, and for CM, RR = 2.28	TMD sign and symptom was 78% for EM and 100% for CM
12	Paolo *et al*., 2017, Italy^28^	Cohort	-	Prevalence of migraine in TMD population was 40.26%
13	Contreras *et al*., 2018, Brazil^29^	Cross-sectional	-	86.55% of patients with painful TMD presented comorbid migraine
14	Nazeri *et al*., 2018, India^30^	Case-control	Myofascial pain TMD subjects had a five times chance of developing headache and those TMD patients with anxiety or depression had more chance of developing migraine	-
15	Ashraf *et al*., 2019, Finland^31^	Cross-sectional	Painful muscular TMD was associated with the presence of migraine (OR = 1.5)	Overall TMD patients reported 14.58% of migraine. Muscular disorder in migrainous patients was 81.25%
16	Fernandes *et al*., 2019, Brazil^32^	Case-control	Painful TMD group was likely to have migraine (OR = 3.0)	Prevalence of migraine in painful TMD was 46.99%, non-painful TMD was 22.72%
17	Wieckiewicz *et al*., 2020, Poland^33^	Cross-sectional	Patients suffering from TMD- related pain were likely to have migraine (OR = 4.53)	Prevalence of migraine in pain - related TMD group was 31.93%

## Epidemiology

TMD pain was observed in patients with primary headache; a population study reported 27% of headache people (any primary headaches) experienced TMD pain, while only 15% of the non-headache group suffered from TMD pain. Also, the prevalence of headache was noticeably higher for the TMD group (72%) than the control group (31%).^34^ Migraine, particularly, is the most prevalent headache in TMD population (55%), followed by tension-type headache (30%).^20^ The odds ratio for migraine in those with TMD was 2.76, which represents that individuals with TMD are two times more likely to develop migraine greater than those without TMD condition.^20^ Moreover, an increased number of TMD symptoms was associated with higher prevalence of migraine headache and chronic daily headaches.^18^

The OPPERA study revealed that migraine and frequent headaches are a significant risk factor for the development of first-onset TMD symptoms.^35^ The headache prevalence and frequency elevated during the observation period among patients who developed TMD. Interestingly, the most outstanding change is that the prevalence of definite migraine episodes increased ten-fold in the TMD group. The result of this study suggested another aspect of relationship between TMD and migraine in that migraine *per se* might also be an exacerbating factor for TMD.^35^

From the published studies, the evidence on the influence of TMD on migraine progression or chronicity is limited and inconclusive. A previous study explored the role of TMD in the development of migraine and reported that frequent symptoms of TMD and frequent headache were strongly associated (odd ratio = 4.1), suggesting that TMD might be a potential factor to induce chronic migraine.^36^ Furthermore, Bevilaqua *et al.* has reported that migraine patients with TMD are 2-3 times more likely to experience cutaneous allodynia (pain occurs due to non-painful stimuli).^37^^,^^38^ Migraine pain, however, can cause not only pain in maxillary (V2) and mandibular (V3) nerve areas, but also allodynia, a source of accompanying discomfort and pain that is probably misdiagnosed as TMD.^39^

## Relationship between TMD and migraine

A growing body of research has established several biopsychosocial factors associated with increased risk for the development and persisting TMDand migraine.^40^^,^^41^^,^^42^^,^^43^^,^^44^ It is therefore important to recognise that various biopsychosocial factors, including shared physiology, psychological behaviours and environmental factors, may as well contribute to the relationship between them. While this review primarily focuses on the anatomical and physiological aspects of the relationship between TMD and migraine, it is essential to acknowledge the potential influence of other comorbid pain conditions and psychological variables during the diagnostic process.

### Anatomical and clinical perspectives

Migraine pain commonly occurs in the ophthalmic branch of the trigeminal nerve (V1) and occasionally radiates to the V2 and V3 branch.^45^^,^^46^^,^^47^ Isolated facial pain in migraine, that is, pain occurring only in the V2 or V3 region, is rare^48^ and is recently classified by the ICOP as isolated orofacial migraine.^11^ During the onset or attack of migraine, many patients experience spontaneous pain in the teeth, cheek, masticatory muscles and periauricular region. Such these presentations of migraine can understandably be misdiagnosed as odontogenic pain, sinusitis or TMD.^25^^,^^49^^,^^50^ This misdiagnosis also led to inappropriate treatment as evidenced by case reports of migraine patients being mistakenly treated as toothaches, resulting in tooth extractions, which unsurprisingly do not improve the pain.^51^^,^^52^

Similar to the distribution of migraine pain, TMD pain can radiate widely in the orofacial and cranial regions.^53^ A cross-sectional study found that the incidence of undiagnosed TMD in headache patients was 25%.^54^ Among primary headaches, the incidence of TMD was highest in migraine patients compared with the other diagnoses.^54^ This could possibly suggest that TMD pain could be the cause of pain in some patients with headaches, given the close anatomical relationship between the muscles of mastication, TMJ and the head.

## Pathophysiological perspectives

In addition to the anatomical overlap between TMD and migraine, a growing number of studies have demonstrated the relatedness of these disorders in terms of pathophysiological mechanism.

### Peripheral and central sensitisation

One of the possible links between migraine and TMD is peripheral and central sensitisation.^34^ When peripheral injury triggers pain signals in the trigeminal nerve, local tissue inflammation releases cytokines and pro-inflammatory mediators that stimulate and amplify the pain response. Peripheral sensitisation decreases the depolarisation threshold so that normal stimuli are perceived as painful. The persistence of peripheral pain input can lead to central sensitisation, resulting in increased excitability of central pain pathways. Central sensitisation is the physiological hallmark of persistent pain syndromes and is responsible for the clinical symptoms of hyperalgesia and allodynia.^46^ Therefore, normal or sub-threshold stimulators from TMJ and associated structures may become migraine-inducing factors or *vice versa*.

A prominent example of peripheral input from both conditions might be the occurrence of myofascial pain. The involvement of myofascial mechanisms in migraines have been reported.^55^^,^^56^^,^^57^^,^^58^^,^^59^^,^^60^ However, the exact pathophysiological relationship between myofascial pain and migraines remains unclear. Myofascial trigger points (MTP) can be classified into active and latent trigger points.^61^ Active trigger points cause constant pain, while latent trigger points only elicit pain when manually palpated.^61^ It has been hypothesised that sustained muscle contraction in MTP leads to hypoxia and ischemia, resulting in increased concentrations of inflammatory mediators, such as calcitonin gene-related peptide (CGRP) and substance P.^58^ It seems that MTP in the muscle of mastication is common in both conditions, myofascial TMD and migraine. Although these studies have examined MTP in multiple muscles of the head and neck, it is still uncertain which specific muscles are most affected.^62^

Several studies have showed a high occurrence of active and latent MTP in individuals with migraine, both during and after a migraine attack.^56^^,^^57^^,^^59^ Additionally, palpating these MTP can even induce a migraine attack in some migraineurs.^56^^,^^63^ This indicates that individuals with migraines may experience ongoing myalgia even after their migraine attacks have subsided. Consequently, there is a possibility of misdiagnosing muscle-related TMD in migraineurs who are currently not exhibiting symptoms of migraines at the time.

The myofascial mechanism in migraines can potentially be explained by both a bottom-up and a top-down model.^62^^,^^64^ In the bottom-up model, peripheral nociceptive transmission sensitises the central nervous system, resulting in a lower pain threshold. On the other hand, central sensitisation may contribute to the development of myofascial trigger points. These models may help explain the constant myalgia in migraineurs after a migraine attacks. Although the myofascial trigger points are common in both myofascial TMD and migraine patients, it should not be yet assumed that the presence of masticatory myalgia is directly and solely related to the comorbidity of both conditions.

### Cross-excitation in the trigeminal ganglion

TMD and migraine might trigger each other because of excitation of one trigeminal branch activates the other branch. Although the dura mater is innervated mainly by V1 branch, it is also supplied by the meningeal fibres of branches V2 and V3.^51^^,^^65^ Therefore, the anatomical connection of these fibres may play a role in cross-excitation and explain why migraine pain may occur in the V2 and V3 regions. The peripheral afferents from both meningeal tissues, where migraine attacks occur, and the TMJ apparatus project towards the trigeminal ganglion. Nociceptive input from both intracranial and extracranial tissues converges in the nucleus caudalis and then projects to the thalamus, cortex and limbic system.^66^ Therefore, it can be hypothesised that the nociceptive inputs from the periphery (TMJ and masticatory muscles) could trigger the migraine-associated neurons at the level of the trigeminal nuclei.

Neuromusculoskeletal dysfunction of the cervical spine (C2, C3) has been found to contribute to both TMD and migraine via the trigeminocervical complex (TCC).^67^^,^^68^ TCC is a key pathway that elucidates the relationship between the upper neck, TMJ and trigeminal nerve, which can be associated with a variety of disorders of the neck, face and head. The convergence of the nociceptive pathways of the upper cervical spine and the trigeminal system allows pain signals from the neck to be referred to the trigeminal sensory receptive fields in the face and head. Thus, not only is migraine a comorbidity of TMD, but neck pain and cervicogenic headache may be a non-skippable disorder that should be considered in TMD treatment.

### Molecular link

The release of proinflammatory molecules could play a role in the sensitisation and association between TMD and migraine. In particular, the level of CGRP are significantly higher in TMD patients when compared to healthy controls, and the magnitude of CGRP levels correlates positively with pain intensity.^68^ CGRP is released from trigeminal fibres and is the most potent known peptidergic dilator of peripheral and cerebral blood vessels and an important factor in the development of neurogenic inflammation and migraine.^69^ CGRP receptors are widely expressed in the trigeminal system. Therefore, it is possible to hypothesise that the expression of CGRP in the trigeminal ganglion because of TMD pathology could stimulate the release of pro-inflammatory mediators and then cause activation of the intra-trigeminal ganglion, leading to excitation of peripheral and meningeal afferents and the development of migraine. However, the relationship between the two diseases is potentially dynamic. We can suggest that the increase in CGRP levels during migraine attacks can also trigger and worsen TMD symptoms.

## Differentiation of TMD and migraine

The aim of the assessment is to determine the underlying cause of TMD pain, distinguishing between cases in which TMD is comorbid with migraine and cases in which migraine pain mimics TMD. One of the key features of migraine presentation (within or without an actual attack) is sensitivity to light, noise and other sensory stimuli (Table 2). When patients present with TMD symptoms, apart from investigation by following the TMD diagnostic criteria, assessment of headache history is essential for clinician to avoid overlooking underlying migraine.

**Table 2:** Migrainoid features

Pattern of headache	Pulsating headache
	Moderate to severe pain intensity
Attack duration	4-72 hours
Aura: temporary disturbances before the migraine pain	Visual sign, speech or language difficulty
	Muscle weakness, numbness or tinging sensation on the face and limbs
Accompanying symptoms	Nausea and/or vomiting
	Sensitivity to light, sound or smell
Physical activity	Pain gets worse with physical movement
Cutaneous allodynia	Pain when hair brushing, head massage, wearing eyeglasses, shaving, exposure to heat or cold
Cranial autonomic features	Lacrimation, conjunctival injection, facial swelling, rhinorrhoea, nasal congestion, ptosis

Migrainous symptoms can be assessed by using a self-answer questionnaire: the ID-migraine item.^70^ The questionnaire facilitates rapid detection of migraine in primary care setting and it consists of three screening questions that inquire about headache-related disability, nausea and sensitivity to light in the past three months (Appendix 2). The ID-migraine indicates the presence of migraine if a patient answered ‘yes' to at least two of the three questions. Diagnostic accuracy revealed a sensitivity of 0.81, a specificity of 0.75, and a positive predictive value (PPV) of 0.93. The three-item ID migraine questionnaire then also proved beneficial for detecting migraine in patients with TMD and orofacial pain.^71^ The diagnostic values showed a sensitivity of 0.58, a specificity of 0.98, and a PPV of 0.93. Interestingly, Kim's study showed the same PPV (93%) as the original study. Hence, the ID-migraine questionnaire can be a quick, applicable and valuable tool for dentists to screen patients with headache symptoms in their clinical setting. For more generic headache screening and monitoring, the HIT-6 is advised to assess the impact of headache but would not act as a diagnostic aid specific to migraine.^72^^,^^73^

**Fig. 1:**
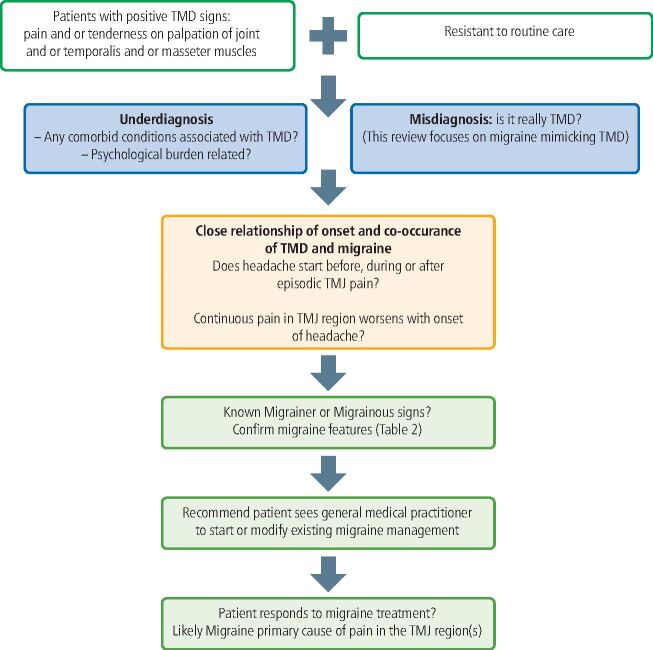
Diagnostic scheme for excluding migraine in patients with TMD symptoms

Regarding the clinical examination for diagnosing TMD, the International Network for Orofacial Pain and Related Disorders Methodology has provided comprehensive tools.^74^ While the reproducible pain on TMJ and masticatory muscle palpation plays a crucial role in diagnosing TMD, it is important to be cautious of myofascial pain among individuals with migraines, as mentioned previously. It is worth noting that even if migraine symptoms are not observed, there is still a possibility of misdiagnosing painful TMD based on muscle palpation. Thus, a comprehensive approach that includes a thorough history interview and assessment of clinical findings becomes imperative in identifying the potential cause of symptoms. We suggest a simple consideration to exclude migraine in patients with positive TMD symptoms (Fig. 1). This approach would benefit those patients with either atypical TMD symptoms or those who have failed multiple TMD treatments. If migraine is the fundamental drive behind the patient's presentation with TMD signs and remains unaddressed, then treatment will inevitably fail. Once the optimal diagnosis is made, there are helpful treatment guidelines for clinicians to ensure that proper treatment is appropriately provided, such as the National Institute for Health and Care Excellence guideline for headache management^75^ and the clinical pathway for adults with headache and facial pain by the National Neurosciences Advisory Group.^76^

## Headache or migraine attributed to TMD

This review earlier outlines that migraine sometimes behaves similarly to TMD pain; on the contrary, TMD can also induce headache and it is recognised as headache attributed to TMD (HATMD) according to the classification of DC/TMD^10^ and ICHD-3^12^ (Appendix 3). The confirmation of familiar headache when jaw movement or muscle palpation during clinical examination is key for identifying HATMD. Individuals with migraine pain who are found to have TMD pain as the original source of migraine should be diagnosed as having migraine attributed to TMD, and solo TMD treatment, not additional headache treatment, may be sufficient to improve the headache. Published evidence of this is found in one study of patients with HATMD who underwent TMD treatment only. The result showed a significant correlation between a reduction in TMD symptoms and decrease in the frequency and intensity of headaches.^77^ On the other hand, if the TMD symptoms originate from migraine, as described above, a single TMD treatment will not benefit these patients. Therefore, treatment response may be a hint to helping the physician determine the true cause of the pain condition.

HATMD is common among patients with chronic myogenous TMD.^77^ Headaches often present as migraine^77^ and are associated with higher headache frequency and familiar masticatory muscle pain responses on examination.^78^ The study has shown that evoked pain on clinical examination was a crucial diagnostic component of HATMD, already included in the diagnostic criteria for HATMD.^78^ Headaches originating from TMD may have several causes: pain in the temporalis muscle (the muscle located in the temple area) or pain in other masticatory muscles or in the TMJ radiating to the temporal region. To ensure appropriate and comprehensive management, it is necessary to determine the underlying condition, such as primary headache, cooccurring with TMD. The ambiguity between HATMD, especially tension-typed headache and myalgia of the masseter muscles, has been discussed.^79^ The site of pain (temporal region) between the two diagnoses largely overlapped. Most people were diagnosed with HATMD along with myalgia of the temporalis muscle. Therefore, they suggested that HATMD and myalgia of the temporalis muscle could be considered as a single clinical entity. As migraine can be initiated by the onset of any pain within the trigeminal system (for example toothache), this may further undermine HATMD being a single clinical entity.^80^

## Conclusion

TMD and migraine are prevalent and highly inter-related. Distinguishing TMD and migraine can be challenging, especially when both disorders co-exist in a single individual. Understanding the relationship between TMD and migraine is essential and using a simple structured pain history, including headache, would improve the recognition of pain comorbidity and determine whether the patient has migraine mimicking TMD or whether it is TMD in isolation. The crucial message is that it is essential to exclude migraine and other primary headaches that can potentially cause or exacerbate TMD symptoms, thus ensuring that the patient receives appropriate treatment. In addition, it is essential to recognise when TMD may precipitate or worsen ongoing headaches, which requires a dual approach to patient care by optimising TMD care and headache management. Moreover, the need for specific studies on the relationship between the two conditions may encourage clinical researchers to conduct further studies to prove the link between TMD and migraine headaches and provide more effective treatment.

**Table:** Appendix 1 Diagnostic criteria for the most two common pain-related TMDs (ICOP classification)

**Muscle-related pain**
**Primary myofascial orofacial pain** Pain in masticatory muscles, with or without functional impairment, not attributable to another disorder.
A. Myofascial pain fulfilling criteria B-D B. Occurring in one or more episodes,* or unremitting C. Reported in the jaw, temple, ear and/or in front of ear, with both of the following: 1. confirmation on examination of location(s) in the temporalis and/or masseter muscle(s) 2. provoked by either or both of: a. palpation of the temporalis and/or masseter muscle(s) b. maximum unassisted or assisted jaw opening movement(s) D. Modified** by jaw movement, function or parafunction (eg tooth-grinding or clenching) E. Not better accounted for by another ICOP diagnosis.
**TMJ-related pain**
**Primary temporomandibular joint pain** Pain localised to the TMJ, occurring at rest or during jaw movement or palpation, with no known causative disorder. The diagnosis corresponds fully to the DC/TMD diagnosis temporomandibular joint pain.
A. Pain in and/or in front of the ear(s), fulfilling criteria B-D B. Occurring in one or more episodes,* or unremitting C. Both of the following: 1. confirmation on examination of location in the area(s) of one or both temporomandibular joint(s) 2. provoked by either or both of: a. palpation of and/or around the lateral pole(s) of the mandibular condyle(s) b. maximum unassisted or assisted jaw opening, right or left lateral and/or protrusive movement(s) D. Modified** by jaw movement, function or parafunction (eg tooth-grinding or clenching) E. Not better accounted for by another ICOP diagnosis.
Key: * = Episodes may be single or recurrent within any day, each lasting at least 30 minutes and with a total duration within the day of at least two hours ** = Pain may be increased or decreased.

**Table:** Appendix 2 Diagnostic criteria for migraine

Migraine without aura*	Migraine with aura*
A. At least five attacks fulfilling criteria B-D B. Headache attacks lasting 4-72 hr (untreated or unsuccessfully treated) C. Headache has at least two of the following four characteristics: 1. Unilateral location 2. Pulsating quality 3. Moderate or severe pain intensity 4. Aggravation by or causing avoidance of routine physical activity (eg walking or climbing stairs) D. During headache at least one of the following: Nausea and/or vomiting Photophobia and phonophobia E. Not better accounted for by another ICHD-3 diagnosis.	A. At least two attacks fulfilling criteria B and C B. One or more of the following fully reversible aura symptoms: 1. Visual 2. Sensory 3. Speech and/or language 4. Motor 5. Brainstem 6. Retinal C. At least three of the following six characteristics: 1. At least one aura symptom spreads gradually over ≥5 minutes 2. Two or more aura symptoms occur in succession 3. Each individual aura symptom lasts 5-60 minutes 4. At least one aura symptom is unilateral 5. At least one aura symptom is positive 6. The aura is accompanied, or followed within 60 minutes, by headache D. Not better accounted for by another ICHD-3 diagnosis.
**Chronic migraine**	ID-migraine (the ID-migraine validation study)**
A. Headache (migraine-like or tension-type-like) on ≥15 days/month for >3 months, and fulfilling criteria B and C B. Occurring in a patient who has had at least five attacks fulfilling criteria B-D for ‘1.1 Migraine without aura' and/or criteria B and C for ‘1.2 Migraine with aura' C. On ≥8 days/month for >3 months, fulfilling any of the following: 1. Criteria C and D for ‘1.1 Migraine without aura' 2. Criteria B and C for ‘1.2 Migraine with aura' 3. Believed by the patient to be migraine at onset and relieved by a triptan or ergot derivative D. Not better accounted for by another ICHD-3 diagnosis.	**Screening question** During the last three months, have you ever had any of the following symptoms with your headache? 1. Do you feel nauseous or sick to your stomach when you have a headache? 2. Does the light bother you when you have a headache? 3. Has your headache ever limited your ability to work, study or our activities for a day? Answering ‘yes' to two or more of these questions, patient is positive to have migraine with a sensitivity of 0.81, a specificity of 0.75 and positive predictive value of 0.93.
“Key: * = The diagnostic criteria by ICHD-3 ** = The screening questions for migraine diagnosis from the ID-migraine validation study by Lipton et al, 2003”

**Table:** Appendix 3 Diagnostic criteria for headache attributed to TMD

ICHD-3*	DC/TMD**
A. Any headache* fulfilling criterion C B. Clinical evidence of a painful pathological process affecting elements of the temporomandibular joint(s), muscles of mastication and/or associated structures on one or both sides C. Evidence of causation demonstrated by at least two of the following: 1. The headache has developed in temporal relation to the onset of the temporomandibular disorder, or led to its discovery 2. The headache is aggravated by jaw motion, jaw function (eg chewing) and/or jaw parafunction (eg bruxism) 3. The headache is provoked on physical examination by temporalis muscle palpation and/or passive movement of the jaw D. Not better accounted for by another ICHD-3 diagnosis**	Headache in the temple area secondary to pain-related TMD (see note) that is affected by jaw movement, function, or parafunction, and replication of this headache occurs with provocation testing of the masticatory system.
	**History** Positive for both of the following: A. Headache^†^ of any type in the temple AND B. Headache modified with jaw movement, function, or parafunction. **Examination** Positive for both of the following: A. Confirmation^††^ of headache location in the area of the temporalis muscle(s) AND B. Report of familiar headache‡ in the temple area with at least one of the following provocation tests: 1. Palpation of the temporalis muscle(s) OR 2. Maximum unassisted or assisted opening, right or left lateral, or protrusive movement(s). Validity sensitivity 0.89; specificity 0.87.
**Note:** 1. Usually temporally located on one or both sides. 2. There is some overlap between 11.7) Headache attributed to TMD arising from muscular tension and 2) Tension-type headache. When the diagnosis of TMD is uncertain, the headache should be coded as 2. Tension type headache or one of its types or subtypes (presumably with pericranial muscle tenderness)^‡^ **Comments:** 11.7: Headache attributed to TMD is usually most prominent in the temporal region(s), preauricular area(s) of the face and/or masseter muscle(s). It may be unilateral but is likely to be bilateral when the underlying pathology involves both temporomandibular regions. Pain referral to the face is common; after tooth pain, TMD is the most common cause of facial pain. Pain generators include disc displacements, joint osteoarthritis, degenerative disease and/or hypermobility, and regional myofascial pain. Diagnosis of TMD can be difficult, with some controversy regarding the relative importance of clinical and radiographic evidence. Use of the diagnostic criteria evolved by the International RDC/TMD consortium network and orofacial pain special interest group is recommended.	**Comments:** The headache is not better accounted for by another headache diagnosis. **Note:** A diagnosis of pain-related TMD (eg, myalgia or TMJ arthralgia) must be present and is established using valid diagnostic criteria.
Key: * = The ICHD-3 ** = DC/TMD † = The time frame for assessing pain including headache is in ‘the last 30 days' since the stated sensitivity and specificity of these criteria were established using this time frame. Although the specific time frame can be dependent on the context in which the pain complaint is being assessed, the validity of this diagnosis based on different time frames has not been established. †† = The examiner must identify with the patient all anatomical locations that they have experienced pain in the last 30 days. For a given diagnosis, the location of pain induced by the specified provocation test(s) must be in an anatomical structure consistent with that diagnosis. ‡ = ‘Familiar pain' or ‘familiar headache' is based on patient report that the pain induced by the specified provocation test(s) has replicated the pain that the patient has experienced in the time frame of interest, which is usually the last 30 days. ‘Familiar pain' is pain that is similar or like the patient's pain complaint. ‘Familiar headache' is pain that is similar or like the patient's headache complaint.	
